# Time Spent on School-Related Activities at Home During the Pandemic: A Longitudinal Analysis of Social Group Inequality Among Secondary School Students

**DOI:** 10.3389/fpsyg.2021.705107

**Published:** 2021-08-11

**Authors:** Sabine Zinn, Michael Bayer

**Affiliations:** ^1^Socio-Economic Panel (SOEP), German Institute for Economic Research (DIW), Berlin, Germany; ^2^Departement of Social Sciences, Humboldt University, Berlin, Germany; ^3^Leibniz Institute for Educational Trajectories (LIfBi), Bamberg, Germany; ^4^Evangelische Hochschule Nürnberg, Nuremberg, Germany

**Keywords:** educational inequalities, school closures, learning time, secondary-age children, parental education

## Abstract

Substantial educational inequalities have been documented in Germany for decades. In this article, we examine whether educational inequalities among children have increased or remained the same since the school closures of spring 2020 due to the COVID-19 pandemic. Our perspective is longitudinal: We compare the amount of time children in secondary schools spent on school-related activities at home before the pandemic, during school closures, and immediately after returning to in-person learning. We operationalize family socio-economic status using the highest parental educational attainment. Based on the theoretical assumption that the pandemic affected everyone equally, we formulate a hypothesis of equalization during the first period of school closures. For the period thereafter, however, we assume that parents with a low level of education had more difficulties bearing the additional burden of supervising and supporting their children’s learning activities. Thus, for that period, we postulate an increase in educational inequality. To study our hypotheses, we use data from the 2019 wave of the SOEP and the SOEP-CoV study, both of which are probability samples. The SOEP-CoV study provides a unique database, as it was conducted during the lockdown of spring 2020 and in the following month. For statistical analysis, we use probit regressions at three measurement points (in 2019, in 2020 during the school closures, and in the month after closures). The comparison of these three time points makes our analysis and findings unique in the research on education during the COVID-19 pandemic, in particular with regard to Germany-wide comparisons. Our results confirm the hypothesis of equalization during the first school closures and the hypothesis of an increase in educational in the subsequent period. Our findings have direct policy implications regarding the need to further expand support systems for children.

## Introduction

Parents, children, and schools were largely unprepared when the COVID-19 pandemic reached Germany in 2020, leading to school closures in the spring and again in winter of 2020 and again in spring of 2021 in some parts of Germany (depending on the regional regulations related to incidence levels). The complete closure of schools was an entirely new situation, especially for parents, who were suddenly faced with the challenges of managing and monitoring their children’s school-related activities.

Overall, the pandemic can be understood as a *collective critical life event*. People worldwide, and thus also in Germany, have been affected, although to different degrees depending on the government measures implemented to contain the pandemic and their effects on distinct population groups. One group that has been uniquely affected by containment measures is that of school-aged children and their families. Since spring 2020, a great deal of research has been done on how social inequality plays out in this context (e.g., Blundell et al., 2020; Reimer et al., 2021). However, there are only a few studies that have used representative data to analyze the effects of school closures and the shift of all school-related activities into the home and family context.

Alongside this emerging body of research, there has been ongoing public discussion about the impact of school closures and distance learning on underprivileged children (whose parents have no education beyond secondary schooling) and children living in precarious circumstances (e.g., whose parents are unemployed). These discussions have raised questions related to broader and long-standing debates about educational justice. The United Nations (2020), for example, expects that learning losses due to the pandemic will be immense and that differences between socio-economic groups will (further) widen.

This paper focuses on the impact of school closures on the school-related activities of children in different socio-economic groups. To distinguish group differences arising directly from the “school closure” event from pre-existing differences, we compare students’ school-related activities during the school closures of spring 2020 with their activities in 2019. A further comparison with the period directly following the reopening of schools in spring 2020 provides an impression of the possible persistence of group differences. In our analyses, we focus only on secondary schools. The mechanisms and effects of school closures are likely to be different for children in primary schools and therefore require separate analysis.

## Background and Theory

As one consequence of the sudden school closures, there was a rapid shift from in-person learning in schools to distance learning at home. Some schools were better prepared for this situation than others, especially with respect to computers and digital resources. Early research results (e.g., Huebener et al., 2020) suggest that private schools switched more quickly than public schools from in-person to distance learning. Previous studies have shown that students in private schools are significantly more privileged on average (in terms of socio-economic backgrounds) than students in public schools (Klemm et al., 2018). Their better access to digital educational resources created a kind of Matthew effect (and thus educational inequality): More privileged students are therefore more likely to attend better-equipped schools.

Moreover, it can be assumed that the pandemic has had additional effects that reinforce existing educational inequalities across socio-demographic groups. This assumption results from research on the “summer learning gap” identified in the United States (e.g., Cooper et al., 1996) and on the influence of summer holidays on skills development in school children (e.g., Alexander et al., 2007). Studies have found differences between children of parents with tertiary education and children of parents with secondary education or lower in the amount of time spent on school-related activities (e.g., Lundborg et al., 2014). Andrew et al. (2020) analyzed the amount of time school children in the United Kingdom spent on school-related activities during the lockdown and compared the results with the situation in 2014–15. They found that the children spent an average of 4.15 h per day on school-related activities during lockdown compared to 6.59 h per day in 2014–15. They report that differences in time use on school-related activities between students from different socio-economic groups (measured by annual family gross income) increased in primary schools the longer these schools were closed. This presents a contrast to the situation of children in secondary schools: Here, inequalities persisted during school closures, but did not increase. The results of Grewenig et al. (2020) paint a different picture for Germany. They indicate an average reduction of school-related learning time by about half during the lockdown, with a significant difference between low- and high-performing students, but without significant correlation with parental education. Likewise, Agostinelli et al. (2020) report a dramatic widening of the educational inequality gap between children from poor neighborhoods and children from richer neighborhoods in the United States as a result of the pandemic. They explain their finding by the fact that the former are less likely to benefit from positive peer contacts in the pandemic situation, and their parents are less likely (able) to work from home. Similarly, Haeck and Lefebvre (2020) predict for Canada an increase at about 30% in the socio-economic skills gap (measured using PISA data from 2000 to 2018) caused by the pandemic and the closure of schools during the crisis.^1^ In summary, there is as yet no consistent evidence on how school closures during the pandemic affected educational inequality across different socio-economic groups.

There are two main theoretical perspectives explaining educational inequality between socio-economic groups in general. The first one explains differences in school-related activities from a family investment perspective (e.g., Becker, 1981). This resource-based perspective assumes that resource-rich parents invest more time in supporting and monitoring their children’s school-related activities to compensate for the loss of time caused by school closures. The second theoretical perspective describes educational processes as kind of struggle for relevant cultural capital between different social classes, in which educational investment strategies are an expression of class-specific educational orientations (see Bourdieu, 1984). Both perspectives can be used to explain group- or class-specific differences under normal conditions when the institutional setting is fairly stable and when parents have some idea what to expect from schools and teachers. The pandemic situation is, however, characterized by a high degree of uncertainty around learning, both on the part of schools and teachers and on the part of children and their parents, irrespective of the socio-economic group or social class to which they belong.

These considerations lead to two possible scenarios. The first one refers to the results from studies on the learning gap during long holiday periods, hereafter referred to as *inequality acceleration scenario*. Under this scenario, we expect existing inequalities between groups of secondary school children distinguished according to their socio-demographic characteristics to increase during school closures. Here we adopt a cultural capital perspective, following Ditton et al. (2019) and Sari et al. (2021). Accordingly, we postulate that families’ cultural resources relating to education, such as parental educational attainment, are one of the driving forces behind differences in school-related activities. The second scenario, which we call the *equalization scenario*, is based on the idea that in a situation of high uncertainty on the part of parents and students about what school and teachers will do, there is little or only a moderate impact of educational background on learning behavior (since everyone is affected in the same way). Unlike, for example, the summer holiday effect, where families have some expectation of what will happen next, the pandemic confronts all families with a similar situation of uncertainty. While passing to the next grade can be seen as dependent on the cultural resources of the home (e.g., Lee and Bowen, 2006), during the pandemic all parents are in a situation where they cannot predict what exactly the policy or the school will do next.

It is well known that the degree of parental control is lower in older children than in younger ones (Seydlitz, 1991). This fact is likely to remain unaffected by the pandemic situation. It can therefore be assumed that older students reduce the amount of time they spend on learning activities at home more than younger students during school closures.

Up to this point, we have only discussed the comparison of educational inequalities before and during the period of school closures. But what happens when schools reopen? How long can schoolchildren and parents compensate for the lack of in-person learning before negative impacts begin to appear? To answer these questions, it is important to consider not only the educational capital of parents or families but also parents’ working conditions. Since individuals with low educational capital (i.e., low educational attainment) are more likely to work in jobs that they cannot do at home (e.g., Möhring et al., 2020), it is plausible that parents in this group are not able to sustain their investment in managing and monitoring their children’s school-related learning activities (for an extended period). It is also plausible that once schools reopen, parents with low educational capital will hand more of the responsibility for their children’s learning back over to teachers and schools than other parents, leading to a greater decline in their investment in their children’s school-related activities at home. This assumption is in line with Lareau’s (2000) findings on the relationship between different social classes and schooling, showing that parents with lower levels of education are less able to coordinate their children’s learning activities at home with the school curriculum than other parents. This is confirmed by O’Sullivan et al. (2014), who show that the quality of parents’ help differs significantly between social groups.

Against this background, we expect different effects of the socio-economic background on the time children spend at home on school-related activities during and after school closures. During the period of school closures, we assume that the concrete support provided by schools is more relevant than the educational capital of the parents for children’s learning activities at home. Thus, in this period, we expect that the pandemic works in a more equalizing direction (*equalization scenario*). However, we also expect that the longer the pandemic situation lasts, the more parental investments decrease, especially those of parents with lower educational attainment, so that inequalities existing in combination with inequalities in educational background accelerate further (*inequality acceleration scenario*).

### Hypothesis

Based on the aforementioned theoretical considerations and the idea that the collective event of school closures was unprecedented, meaning that no one had any experience with such a situation before the pandemic, we do not expect any additional effects of educational background on learning behavior during the period of school closures (*Hypothesis 1*). Nevertheless, we expect effects after this phase (*Hypothesis 2*): Concretely, we hypothesize that children whose parents had very low educational attainment fell behind their peers when schools reopened. We consider it plausible that support from teachers and schools during school closures and especially the use of digital resources had a high impact on children’s school-related activities, since digital technologies ensure effective delivery of learning materials to students (*Hypothesis 3*). Finally, we expect that during the school closures, older students spent less time on learning at home than younger students (*Hypothesis 4*).

## Data and Methods

### Data

We studied home learning time of students in secondary education by comparing data from three time periods: the period before the COVID-19 pandemic (spring 2019), the period during the first lockdown (April-May 2020), and the period shortly after the first lockdown came to an end (May-June 2020). Data for the pre-pandemic period come from the 2019 annual wave of the German Socio-Economic Panel (SOEP) study. Data for the lockdown period and the period shortly thereafter stem from a special survey (SOEP-CoV) of SOEP respondents on their living conditions during the pandemic, conducted from April 1 to the end of June 2020. The 2019 SOEP wave was conducted mainly in CAPI, meaning that interviewers visited respondents’ households and asked all adult household members a variety of questions on socio-economic and psychological topics, such as their own and their household’s financial situation, marital status, and personal well-being. Adolescents, children, and their parents completed special questionnaires for these age groups, including questions about school and the learning situation at home. In the SOEP-CoV study, all households with a valid telephone number (except for the refugee samples) were contacted by phone (in CATI), and one adult member of the household was asked to participate in the survey. The SOEP-CoV sample was randomly divided into nine cross-sectional tranches of participants who were contacted at 2–3 week intervals (for details, see Kühne et al., 2020). A question about how much time children spent learning at home was part of the questionnaire that went out to tranches 2 to 9. The periods in which they were surveyed (April 14 to May 24) correspond relatively closely to the time period when the German federal states ordered the closure of schools for the first time. The survey periods for tranches 5–9 (May 25 to June 28) cover the 5 weeks thereafter and thus the period when the schools returned to a regular mode of operation. The SOEP-CoV study is designed such that the first four subsamples comprise approximately 75% of the cases, and the remaining 5 tranches comprise circa 25%. The reason is that the study designers wanted to lay as much statistical power as possible to the period of the early period of the pandemic, see Kühne et al., 2020 To ensure that the working population (or, rather, the people who were not working from home) could also be reached, half of the calls were made in the late afternoon or evening (51% in total, see also Siegers et al., 2021).

Our analytic samples relate to secondary school children, aged 10–18 years. The 2019 SOEP wave contains data on *N* = 1,433 secondary school children (with a mean age of 14.1, 49.5% girls and 41.3% attending Gymnasium) and the 2020 SOEP-CoV data contain information on *N* = 1,028 secondary school children (*N* = 723 in the tranches 2–4 with a mean age of 15.3, 48.3% girls, 48.7% attending Gymnasium; *N* = 305 in the tranches 5–9 with a mean age of 15.2, 47.2% girls, 42.6% attending Gymnasium).

The outcome variable is a categorical variable for *time spent per day on school-related activities at home*. The categories are “less than 30 min,” “between 30 min and 1 h,” “between 1 and 2 h,” “between 2 and 3 h,” “between 3 and 4 h,” and “more than 4 h.” In 2019, students themselves reported the time they spent on school-related activities at home, while in 2020, this information was given by their parents (about the youngest schoolchild in the household). These two different reporting perspectives constitute a potential measurement error when comparing the amount of time reported in 2019 and 2020. For example, children might think that they spend more time on schoolwork than their parents perceive to be the case. In order to measure the strength of this effect with regard to our study results, we would need comparable measurement points, i.e., statements from parents and children about time spent on learning that refer to the same time periods. Unfortunately, such information is not available to us. However, asking questions about time use in the form of categories mitigates the problem: The difference between parents and children in their allocation to these discrete categories is likely to be negligible compared to the statistical imprecision associated with the sample and sample size.

A central explanatory variable in our study is the educational level of the parents. In our analyses, we include the *highest educational attainment of the parents living in the household* according to the CASMIN classification scheme as an independent variable with the three ordered categories “low education,” “medium education,” and “high education.” Furthermore, we include the *age of the child* (ages 10–14 or 15–18) in our analysis. We measure *school support* during the 2020 spring lockdown by whether children received learning materials through digital channels (i.e., email or cloud) and also whether multiple channels (i.e., email, cloud, conferencing tools, materials distributed prior to the lockdown, or other means such as in-person meetings with teachers) were used to provide students with learning materials. To capture the potential impact of parental time resources, we also considered *parents’ employment status*, categorized by “at least one parent works full-time or part-time,” “neither parent works,” and “parents are in some other type of employment that is not full-time or part-time, or are without work” (e.g., working reduced hours, or on “short-time work”). As possible confounders, we included the children’s *gender, type of school*, and *performance level* at school. The *type of school* distinguishes Gymnasium from other types of secondary school. Performance at school was measured by very good or good grades in mathematics and German (average grades 1–2) and moderate to poor grades (average grades 3–6). To capture the differences in in-person versus distance learning immediately after the lockdown in spring 2020, we additionally controlled for the type of learning during this period. Here, we distinguished between in-person learning for all or part of the week and distance learning at home.

For the two periods considered in 2020, the data on parents’ employment status, the type of school, and students’ performance levels (i.e., school grades) were taken from the 2019 wave of the SOEP. The reason for this is that the SOEP-CoV questionnaire does not provide (complete) information on these characteristics. The time span between the 2019 SOEP wave and the SOEP-CoV survey is less than 1 year. Therefore, we still consider the information from 2019 to be sufficiently reliable for the type of school a child attended in 2020. The same applies to children’s performance levels. For a large proportion of the children surveyed in 2020, the 2019 SOEP wave did not contain any information on school type or grades in math or German. This is an issue that has to be taken into account in the statistical analysis.

Table 1 shows the weighted sample statistics for the characteristics considered for the three time points studied, i.e., spring 2019, April 14 to May 24, and May 25 to June 28. Post-stratified survey weights for the households in which the children live were used to obtain these figures (see “Methods” section for more details). Overall, the distribution of the independent variables shows the expected pattern.

**TABLE 1:** Sample composition, column %.

	**2019**	**14.4.–24.5.2020**	**25.5.–28.6.2020**
**Time spent on school-related activities at home**			
Less than 30 min	0.19	(0.02)	(0.06)
Between 30 min and 1 h	0.38	0.07	0.19
Between 1 h and 2 h	0.31	0.23	0.30
Between 2 h and 3 h	0.08	0.28	0.20
Between 3 h and 4 h	0.02	0.20	0.12
More than 4 h	(0.00)	0.21	0.13
Information missing	(0.02)	(0.01)	(0.01)
**Highest parental level of education**			
Low (CASMIN 0,1a,1b,2b)	0.09	0.10	(0.11)
Medium (CASMIN 1c,2a,2c)	0.54	0.52	0.52
High (CASMIN 3a,3b)	0.36	0.36	0.35
Information missing	(0.01)	(0.02)	(0.02)
**Employment status of parents in 2019^†^**			
At least one parent working full-time	0.74	0.73	0.73
Neither parent working full-time, at least one part-time	0.15	0.20	0.16
Neither of the parents employed	0.08	(0.04)	(0.06)
Other kind of employment	(0.02)	(0.02)	(0.04)
Information missing	(0.01)	(0.01)	(0.01)
**Age of school child**			
10–14	0.72	0.70	0.70
15–18	0.28	0.30	0.30
**Gender of child**			
Male	0.49	0.52	0.50
Female	0.51	0.48	0.50
**School type**			
Gymnasium^‡^	0.41	0.41	0.43
Other school type	0.58	0.49	0.49
Information missing	(0.01)	0.10	(0.09)
**Performance of school child**			
Grade 1 or 2	0.26	0.21	0.17
Grade 3 or worse	0.63	0.45	0.35
Information missing	0.11	0.34	0.47
**Provision of learning material**			
Digital (email, cloud)	–	0.95	–
Not digital	–	0.05	–
**School support**			
Learning material provided through several channels	–	0.71	–
Only one channel or none	–	0.29	–
**In-person versus distance learning**			
In-person learning all or part of the week	–	–	0.13
Distance learning at home	–	–	0.87
Sample size (unweighted)	1,433	723	305

*Weighted statistics (using post-stratified survey weights on household level). Cells with fewer than 30 observations are given in parentheses. ^‡^Gymnasium: German upper secondary school providing university entrance qualifications. ^†^Employment status of parents measured in 2019 since information for the lockdown was only available for one adult household member (the CATI respondent).*

### Methods

We used a simple descriptive measure: We calculated whether schoolchildren spent more than 2 h per day on school-related activities at home (or less). We chose the cut point of 2 h because the category ‘‘between 2 and 3 h’’ constitutes the weighted median (category) during the lockdown and ‘‘between 1 and 2 h’’ the weighted median (category) directly after the lockdown.^2^ All descriptive analyses were carried out separately for 2019, the 2020 spring lockdown, and for the period shortly thereafter. For each of these time periods, we also conducted binary response analysis (probit regressions) to gain deeper insight into the impact of our study’s focus variables on time use under the specific circumstances (i.e., lockdown or not).

All analyses were weighted using non-response-adjusted and post-stratified survey weights for the households in which the 2019 SOEP and SOEP-CoV respondents live. The weighting strategy used in the annual SOEP survey and the variables considered for related non-response adjustment as well as used for post-stratification are described in great detail in Siegers et al. (2020). The weighting procedure used the SOEP-CoV study is described in detail in Siegers et al. (2021). In the related non-response analyses, we paid particular attention to employment status, income, gender, number of persons in a household, household type, educational level, and migration background. For weighting the SOEP-CoV study, it is crucial to consider whether any adult household member was employed as an essential or frontline worker, as well as the COVID-19 incidence at the NUTS-3 regional level (on the day of the interview). Possible period effects in participation behavior were controlled by interaction terms with the different sample tranches. Post-stratification for the 2019 SOEP wave and the SOEP-CoV survey were based on distributions taken from the 2019 Microzensus for various regional and socio-economic characteristics, including age, gender, household size, citizenship, size of municipality, and federal state. In this study, the weights for the two SOEP-CoV samples (the lockdown sample, i.e., tranches 2–4, and the post-lockdown sample, i.e., tranches 5–9) were further post-stratified to correspond to the proportions of secondary school children in the two age groups “10–14” and “15–18” reported in official school statistics for the school year 2019/2020 (Statistisches Bundesamt, 2020). To assess whether the analytic samples used in this study (i.e., secondary school children in 2019, during the 2020 spring lockdown period, and in the period shortly after) represent random sub-samples of the SOEP and SOEP-CoV sample for which the survey weights were originally derived, we conducted a selectivity analysis. For each analytic sample, we estimated a logistic regression model in which the indicator for membership (or non-membership) in the respective sample was the dependent variable. For this, the entire 2019 SOEP wave formed the base sample for the 2019 sample of secondary school children, and the entire SOEP-CoV sample (all tranches 1–9) formed the base sample for the two 2020 samples. All of the household and individual characteristics described above were the covariates. We found that in all three samples, none of the covariates considered had a significant impact on membership probability in the analytic samples. Therefore, the survey weights derived for the 2019 wave of the SOEP and those derived for the SOEP-CoV sample also fit the analytic samples of this study.

We imputed missing values by using the multivariate imputation by chained equations (mice) algorithm by van Buuren and Groothuis-Oudshoorn (2011), applying classification and regression trees (CART) as the imputation routine. To improve the predictive power of the imputation routine, we used several auxiliary variables in addition to the focal variables of this study (such as the family members’ migration background, family status, employment status of the CATI respondent in 2020, regional information, number of children in the household). As suggested by Kim et al. (2006), we entered survey weights into the corresponding imputation models as explanatory variables. We imputed *m* = 20 data sets with 20 iteration steps in the Gibbs sampler of mice. We checked the convergence and meaningfulness of the estimated imputation models by means of the associated mice diagnostics (e.g., trace plots).

We examined the robustness of our results by conducting robustness checks. To see whether besides parental education also the income situation of the household impacted on learning times, we included the monthly net household income in 2019 as an additional explanatory variable in our models (pre-pandemic mean 4450 EUR, SD 2070 EUR, during the 1st lockout mean 4533 EUR, SD 2135 EUR; post 1st lockout mean 4458 EUR, SD 2394 EUR).^3^ We also investigated whether home office (48% during the pandemic, 45% directly thereafter) and the employment situation of the respondents in spring 2020 (during lockdown in spring 2020: 77% in full- or parttime, 15% non-employed, 8% other kind of employment; directly thereafter: 72% in full- or parttime, 17% non-employed, 10% other kind of employment) have an influence on students’ learning times.

For statistical analysis, we used the software R (version x64 3.6.2). All source code for data preparation, descriptive analysis, and regression analysis is freely available at the GitHub link https://github.com/bieneSchwarze/TimeSpentOnSchoolActivitiesAtHomeDuringPandemic.

## Results

### Descriptive Results

Table 2 shows the proportion of secondary school children who spent at least 2 h per day on school-related activities at home, according to time period (before the pandemic, during the spring 2020 lockdown, immediately after the lockdown). We find that this proportion increases from 9 to 68% during the lockdown in spring 2020. This means that 7.5 times more children fell into that category during than before the pandemic. In the period immediately after the lockdown in spring 2020, this proportion fell to 46%. We identified the observed increases as statistically significant. Corresponding tests were carried out using *t*-tests with *p* < 0.05.

**TABLE 2:** Proportion of children spending at least 2 h per day on school-related activities at home (95% confidence intervals in parentheses).

**Time point**	**Proportion**	**95% confidence interval**
In 2019	0.09	(0.07,0.12)
During 2020 spring lockdown	0.68	(0.62,0.75)
After 2020 spring lockdown	0.46	(0.36,0.57)

*Weighted analysis. Confidence intervals have been derived by basic bootstrap.*

Considering the highest parental education attainment in the household as a relevant dimension of educational inequality (see Table 3) as well, we see that in 2019, the proportion of secondary school children who spent at least 2 h per day on school-related activities at home ranged from 2% among children whose parents had low educational attainment to 8 and 10% for children whose parents had medium and high educational attainment, respectively.

**TABLE 3:** Proportion of children spending at least 2 h per day on school-related activities at home, according to the highest educational attainment of the parents living in the household (95% confidence intervals in parentheses).

**Time point**	**High education**	**Medium education**	**Low education**
In 2019	0.08 (0.05,0.11)	0.10 (0.06,0,14)	0.02 (0.00,0,04)
During 2020 spring lockdown	0.70 (0.61,0.81)	0.66 (0.56,0.75)	0.69 (0.51,0.95)
After 2020 spring lockdown	0.49 (0.29,0.67)	0.53 (0.35,0.69)	0.04 (0.00,0.08)

*Weighted analysis. Confidence intervals (given in parentheses) have been derived by basic bootstrap.*

**FIGURE 1:**
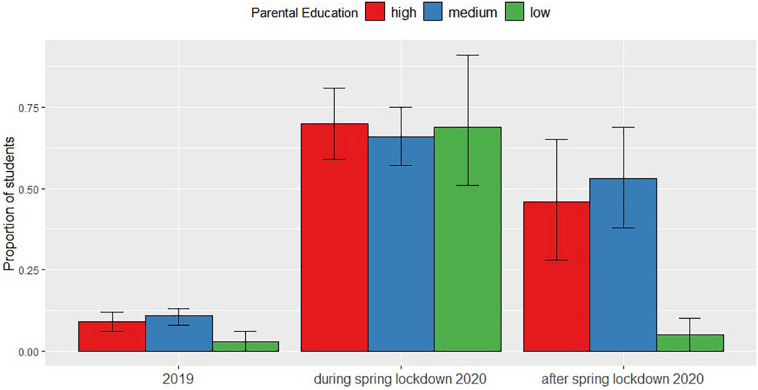
Proportion of children spending more than 2 h per day on school-related activities at home, according to the higher of the parents’ educational attainment. Vertical lines mark 95% confidence intervals. Weighted analysis. Confidence intervals have been derived by basic bootstrap.

This suggests that educational inequality (approximated by the amount of time spent on school-related activities at home) was more pronounced between the group of children whose parents had lower levels of education and those with medium or high levels of education than between the medium and high education groups. During school closures, we find no significant differences between the amount of time spent on schoolwork in relation to the higher of the two parents’ educational attainment. This descriptive result supports our first hypothesis, positing an equalization during the first phase of school closures in Germany. For the period after the lockdown, however, we find that the pre-pandemic differences in home learning times between lower levels of parental education and medium or higher levels of parental education increased dramatically. Compared to children whose parents had a medium or higher level of education, the proportion of children with parents having low educational attainment who spent at least 2 h per day doing school-related activities at home dropped from 69% during the lockdown to 4% in the immediate post-lockdown period. This result supports our second hypothesis regarding an acceleration of educational inequalities due to the pandemic. Figure 1 illustrates this alarming result.

### Regression Results

Table 4 shows the average partial marginal effects from the weighted probit analysis. The model includes all of the aforementioned covariates, as well as the confounders “gender of child,” “school type,” “performance level,” and “type of schooling” (in-person or distance) for the period after the 2020 spring lockdown. These results confirm our descriptive results regarding the influence of parental education levels: During the lockdown, children spent similar amounts of time on schoolwork at home regardless of their parents’ education, but after the lockdown period, in the group of children whose parents had a low level of education, the proportion of children who spent more than 2 h per day at home on schoolwork decreased dramatically and was 47% lower than that of children whose parents had a medium or high level of education (with all other covariates held constant). Both of these results support Hypotheses 1 and 2 (equalization during the first lockdown and inequality acceleration after the lockdown). We see that the use of digital channels for the provision of learning materials has a high impact on children’s school-related activities during the spring 2020 lockdown: The proportion of children who spent at least 2 h per day on school-related activities at home was 39% higher in the group who received learning materials through digital channels than in the group who did not (holding all other covariates constant). This corroborates Hypothesis 3. We find support for Hypothesis 4 as well: The estimated model suggests that older students (aged 15–18) spent less time on school-related activities than younger students (aged 10–14) during the spring 2020 school closure period.

**TABLE 4:** Average partial marginal effects from weighted probit regression.

	**2019**	**14.4–24.5.2020**	**25.5.–28.6.2020**
**Highest parental level of education**			
High (CASMIN 3a,3b)	(Reference category)		
Medium (CASMIN 1c,2a,2c)	0.01	0.03	0.06
Low (CASMIN 0,1a,1b,2b)	−0.07*	0.07	−0.47*
**Employment status of parents in 2019^a^**			
At least one parent works full-time or part-time	(Reference category)		
Neither of the parents employed	0.14*	−0.01	0.17
Other type of employment (e.g., short-time work)	0.07	0.25*	−0.01
**Age of child**			
10–14	(Reference category)		
15–18	0.04*	−0.11*	0.12
**Gender of child**			
Male	(Reference category)		
Female	0.07*	0.00	−0.01
**School type**			
Gymnasium^b^	(Reference category)		
Other school type	−0.01	−0.09	0.24*
**Performance of child**			
Grade 1 or 2	(Reference category)		
Grade 3 or worse	−0.02	−0.04	−0.03
**Provision of learning materials**			
Not digital	(Reference category)		
Digital (email, cloud)	–	0.39*	–
**School support**			
Only one channel or none	(Reference category)		
Learning materials provided through several channels	–	−0.04	–
**In-person versus distance learning**			
Remote learning at home only	(Reference category)		
In person learning all or part of the week	–	–	−0.31*
Pseudo-R^2^ (McFadden)	0.07	0.05	0.15
Sample size	1,433	723	305

*Outcome variable: indicator whether school children in secondary education learned at least 2 h per day at home for school.*

*^∗^p < 0.05.*

*^*a*^Gymnasium: German upper secondary school type providing university entrance qualifications.*

*^*b*^Employment status of parents measured in 2019 since information for the lockdown was only available for one adult household member (the CATI respondent).*

In addition, we find that it has a positive effect on children’s learning time at home if their parents were engaged in another type of employment, such as vocational training or short-time work, during the school closure period. The proportion of children who spent more than 2 h per day on school-related activities at home is 25 per cent higher in this category than among children whose parents were employed full-time or part-time or were unemployed.

Not surprisingly, we see in our results that children who returned to in-person for all or part of the week after lockdown spent less time on school-related activities at home than children who only had distance learning. A nevertheless surprising result is the positive effect on learning time at home in the category “other school type than Gymnasium.” Here, however, it must be taken into account that in most of Germany’s federal states, the post-lockdown period fell exactly in the final examination period for the intermediate school-leaving certificate and the qualified lower secondary school-leaving certificate. This meant that these groups of students had to spend a great deal of additional time studying at home. The final examinations for the Gymnasium (i.e., the Abitur), on the other hand, had already been completed in most cases by this time. We therefore attribute the positive effect detected for other school types (than the Gymnasium) to the final examination period in these school types.

Including the monthly net household income in 2019 as additional variable into our regression analysis did not impact on the results. The related average marginal effect was zero for all three periods studied. Concerning home-office, we see a small but statistically insignificant effect on students’ time use [during 1st lockdown AME = 0.09 with 95% CI (−0.02,0.20); after 1st lockdown AME = 0.09 with 95% CI (−0.09,0.27)]. We also studied whether our results change when including the employment situation of the respondents in spring 2020 instead of their household’s employment situation in 2019. This exchange does not impact on our results [during the 1st lockdown: being not employed as compared to fulltime or parttime employment AME = −0.13 CI (−0.31, 0.06), other type of employment AME = 0.02 95% CI (−0.14,0.18); after the 1st lockdown: being not employed as compared to fulltime or parttime employment AME = −0.01 CI (−0.32,0.29), other type of employment AME = 0.08 95% CI (−0.18, 0.34)]. In conclusion, our results are robust under the assumed model with respect to household’s monthly net income, and the respondents’ employment and home office situation in spring 2020.

## Concluding Remarks

In summary, we found a mixed picture in our study regarding the impact of the spring 2020 school closures due to the COVID-19 pandemic on different groups of secondary school children. In the period of closures, we see a picture of equalization regarding time use on school-related activities at home between children with different parental educational backgrounds. During the closures in spring 2020, all groups, regardless of parental educational background, reduced their school-related learning activities, which at this point was the amount of time normally spent both in school and doing homework or engaging in additional learning activities at home. We find that the few small differences found in learning time can be explained mainly by the teachers or the policies of schools and by the parents’ professional situation and how this affected their ability to work from home. This result is similar to the results of other studies in other countries (e.g., in the United Kingdom; Cullinane and Montacute, 2020; Pensiero et al., 2020). At this point, the pandemic thus had an equalizing effect. For the period immediately after the school closures in spring 2020, children whose parents had a low level of education reduced their learning activities at home substantially compared to children whose parents had medium or higher education. During the school closures, 70% of children spent an average of at least 2 h per day learning at home, regardless of their parents’ education. After this period, this proportion dropped to 4% for children with low-educated parents compared to 53 and 49% for children with medium or highly educated parents. We thus observe an acceleration of inequalities between children of parents with low education and children of parents with medium or high education for the period directly after the closures.

This alarming result undoubtedly has direct policy implications regarding the need to expand support systems for children who are severely affected by educational inequalities. It also raises concerns about the probable massive impact of the second period of school closures in late 2020 and early 2021. If this process of widening learning time gaps continues, the long-term impact on educational inequality could be substantial, if not irreversible. The data we use in our study are not without problems. In particular, we cannot rule out the possibility of measurement error in our dependent variable (students’ time use). Different response behavior among different groups of children (in 2019) or parents (in 2020) could have caused such measurement error–a possible problem that we cannot check with the data at hand. However, the respondents in the study are panelists (in the SOEP) who are used to self-reporting time use in different contexts. This circumstance should at least counteract a possible measurement error.

There are several important questions that we cannot answer with our data. First, we cannot analyze the medium- or long-term effects of differences in learning activities at home on competence development. In fact, only a very few preliminary scenario-based studies on this topic exist at all (Kaffenberger, 2021). The reasons clearly lie in the lack of data that can provide information about future developments. For Germany, data from the National Education Panel in particular offer potential for this type of analysis.

Second, the question remains unanswered how long parents, children, and teachers can compensate for the lack of in-person learning, and what the individual and social consequences will be. In this context, digital teaching can also only be seen as a compensatory measure and not as a solution that should be extended at the current scale into the future. The lack of face-to-face interaction between child and teacher is detrimental first and foremost to children’s psychosocial development (e.g., Haleemunnissa et al., 2021) and is certainly also not conducive to teachers’ well-being and work (e.g., Klapproth et al., 2020; Collie, 2021). While there are preliminary results on the impact of parents’ working conditions during the pandemic on parenting behavior mediated by parental stress (Chung et al., 2020), it is not clear precisely how work-family arrangements and the need to supervise children’s school activities actually affect children’s distance learning at home. Aznar et al. (2021) provide first results on this question for the United Kingdom, and Verweij et al. (2021) for a rather small sample of parents in the Netherlands; however, both studies use non-random samples and therefore do not provide generalizable results. Finally, we do not have enough information in our data about schools and the differences in school policies during and after the school closures in spring 2020 to shed light on school-related effects in our model. This data gap is further complicated by the fact that there were a variety of different strategies used by schools and teachers to deal with the new situation at that time, which are difficult to categorize and thus quantify in a (regression) model. There is high evidence that the fact that we do not see an increase in inequality during the lockdown and the period of distance learning can be explained on the one hand by the equalization scenario (collective event, with an equally high degree of uncertainty for all). On the other hand, we assume that the differences in students’ learning times during the first closure were not significantly caused by the parents’ home or student-specific characteristics, but by the school’s actions and the teachers’ behavior. The different levels of digitalization in the schools probably play a role here, as does the high degree of autonomy of the teachers with regard to planning distance learning.

## Data Availability Statement

The data analyzed in this study is subject to the following licenses/restrictions: The data have been collected as part of a third-funded project. Data usage is therefore restricted. The data will be made available for the scientific community in 2022 with the usual release of the SOEP (German Socio-Economic Panel) annual waves. Requests to access these datasets should be directed to www.diw.de/en/diw_02.c.222516.en/data.html.

## Author Contributions

SZ contributed 60% to this work. MB contributed 40% to this work. Both authors contributed to the article and approved the submitted version.

## Conflict of Interest

The authors declare that the research was conducted in the absence of any commercial or financial relationships that could be construed as a potential conflict of interest.

## Publisher’s Note

All claims expressed in this article are solely those of the authors and do not necessarily represent those of their affiliated organizations, or those of the publisher, the editors and the reviewers. Any product that may be evaluated in this article, or claim that may be made by its manufacturer, is not guaranteed or endorsed by the publisher.

## Footnotes

1 In addition to the studies discussed in detail here, there are a number of other studies that thematically lie in the same thematic field as ours. E.g., Jæger and Blaabæk (2020) examine library borrowing behavior during the period of distance learning with Danish register data and find significant differences with respect to parental educational background.

2 We chose the median time during lockdown and directly thereafter as a compromise to cope with the issue that learning times before the pandemic and learning times during the pandemic are structurally different and cannot be compared one-to-one. In the period before the pandemic, students used the time for school-related activities at home almost exclusively for homework and re-learning the material taught at school. During lockdown periods, all school-related activities took place at home. Shortly thereafter, students were partly taught on site in the school, partly the schooling still took place at home.

3 SOEP data on net household income in 2020 are not yet available at the time of writing this article.
